# Genomic Architecture of Inbreeding Depression Associated With Hatching Failure in an Endangered Parrot

**DOI:** 10.1111/mec.70252

**Published:** 2026-01-21

**Authors:** Yasmin Foster, Stefanie Grosser, Brodie J. Foster, Nicolas Dussex, Aimee Stubbs, Ludovic Dutoit, Theo Atkinson, Fiona Robertson, Ken G. Dodds, Rudiger Brauning, Jeanne M. E. Jacobs, John C. McEwan, Bruce C. Robertson

**Affiliations:** ^1^ Department of Zoology University of Otago Dunedin New Zealand; ^2^ Department of Population Analysis and Monitoring Swedish Museum of Natural History Stockholm Sweden; ^3^ AgResearch Mosgiel New Zealand

**Keywords:** conservation management, hatching success, inbreeding, inbreeding depression, reproductive success, runs of homozygosity

## Abstract

Conservation management of endangered species increasingly relies on genomic approaches to understand how long‐term small population sizes affect the fitness of extant individuals. However, despite the growing investment in genomic resources by conservation programmes, the impact that sequencing methods have on the ability to detect inbreeding‐related phenomena has been largely overlooked. Here, we compare the use of whole‐genome and reduced‐representation sequencing approaches in 148 individuals of the critically endangered parrot, the kākāpō ( *Strigops habroptilus* ), to assess inbreeding and its effects on female reproductive success. We explore how sequencing choice influences the identification of long stretches of homozygosity across the genome (runs of homozygosity, ROH), and compare the conservation implications of the results produced by each method. Both whole‐genome and reduced‐representation sequencing approaches provided comparable estimates of genome‐wide inbreeding (*F*_ROH_) and revealed consistent effects on egg hatching success, suggesting that reduced‐representation sequencing is capable of detecting inbreeding depression in wild populations under certain conditions. Whole‐genome sequencing enabled chromosome‐level inbreeding analyses, which revealed no strong evidence of chromosome‐specific effects beyond the genome‐wide signal. These results suggest that inbreeding depression in kākāpō reflects small effects across many chromosomes rather than strong effects on only a few, and that the primary benefit of whole‐genome sequencing lies in improving the precision of genome‐wide inbreeding estimates rather than identifying chromosome‐specific effects. Our findings highlight the distinct benefits of each sequencing approach in conservation, particularly within the context of resource limitations.

## 1 Introduction

Conservation programmes have long utilised genetic resources to gain insights into the implications of long‐term small population sizes and the recovery potential of endangered species. Recent advancements in genomic sequencing and reductions in cost have led to the emergence of innovative new approaches to understand and promote species recovery processes. Reduced‐representation sequencing methods (RRS), which capture a limited but reproducible fraction of the genome, are an economical approach for generating genomic data across many individuals and are now being widely used in conservation programmes to obtain genome‐wide inbreeding coefficients that inform the management of small and recovering populations (e.g., de Villemereuil et al. 2019; Harrisson et al. 2019). Only a limited number of studies have employed high‐coverage whole‐genome sequencing (WGS) across a large sample of individuals to comprehensively understand inbreeding depression in endangered species (e.g., Hasselgren et al. 2024; Robinson et al. 2019). However, only a few studies have evaluated how the choice of sequencing method can impact estimates of inbreeding in a conservation context (Duntsch et al. 2021; McLennan et al. 2019; Wright et al. 2020). To enable conservation programmes to make better‐informed decisions when investing in genetic resources for endangered and recovering species, additional studies are needed that explore the advantages and limitations of different genomic sequencing approaches for studying inbreeding depression.

Inbreeding depression and associated adverse effects on fitness significantly threaten the recovery of endangered species. Here, we quantify levels of inbreeding, assess their fitness consequences (inbreeding depression), and examine how these effects are distributed across the genome, thereby informing the genomic architecture of inbreeding depression. Studies investigating the relationship between estimates of inbreeding and fitness traits often reveal that highly inbred individuals have significantly lower values for traits relating to reproductive success, such as lifetime breeding success (Duntsch et al. 2023; Willoughby et al. 2019; Zilko et al. 2020) and offspring survival (Hasselgren et al. 2021; Huisman et al. 2016). Advances in high‐throughput sequencing technologies have enabled more accurate assessments of inbreeding and its effects on fitness through a variety of measures of heterozygosity, ranging from allelic variation within microsatellite loci to the increasingly prevalent use of single nucleotide polymorphisms (SNPs) (Hasselgren and Norén 2019; Miller et al. 2014). As the genomic resources of conservation programmes continue to grow and expand in scale (e.g., chromosome‐level reference genomes; Dussex et al. 2021), genome‐wide inbreeding estimates are now becoming routinely used as a way of assessing the overall ‘genetic viability’ of endangered species. The availability of fitness data in such species presents rare opportunities to gain deeper insights into the effects of inbreeding by revisiting previous tests of inbreeding‐fitness correlations using modern genome‐wide estimates of inbreeding, relatedness and genetic load (Dussex et al. 2023), as detailed fitness data are often difficult to obtain in species of conservation concern due to logistical and temporal constraints.

An effective approach to estimate whole‐genome inbreeding is to examine runs of homozygosity (ROH), which represent the proportion of an individual's genome that contains extended stretches of homozygosity resulting from shared ancestry. As an absolute measure of individual autozygosity, the inbreeding coefficient based on ROH is unaffected by the inclusion of specific individuals and is therefore immune to relative estimation biases caused by population allele frequencies (McQuillan et al. 2008). As a consequence, ROH analysis has been shown to provide more precise and less biased estimates of inbreeding when compared to heterozygosity‐based metrics (Caballero et al. 2021; Keller et al. 2011; Nietlisbach et al. 2019). ROH analysis is well suited to investigate several aspects of inbreeding that are informative for conservation management, including the timing and severity of inbreeding in different subpopulations of a species as well as potential mitigation of inbreeding (i.e., breaking up of ROH) resulting from previous management interventions (Kardos et al. 2015). For instance, Humble et al. (2023) found that captive‐managed populations of the scimitar‐horned oryx exhibited a significantly lower proportion of ROH in their genomes and a relative absence of long ROH compared to their unmanaged counterparts. ROH analysis also offers a powerful approach for detecting inbreeding depression in endangered and recovering populations through comparisons of ROH among individuals that vary in fitness‐related measures (Fu et al. 2019; Hasselgren et al. 2021). However, uncertainty remains regarding how accurately RRS datasets can estimate inbreeding using ROH in empirical conservation contexts, despite recent simulation‐based evaluations of method performance and limited empirical applications (Foster et al. 2021; Lavanchy and Goudet 2023). Assessing the impact of ROH on fitness traits across the genome with chromosome‐specific estimates (i.e., inbreeding levels partitioned by chromosome), which consider whether fitness traits are uniformly affected across the genome, may also offer further insights into the genomic architecture of inbreeding depression (Hewett et al. 2024).

The kākāpō ( *Strigops habroptilus* ) is a critically endangered parrot endemic to New Zealand, with a current population of 237 individuals (as of December 2025) mostly managed on offshore islands. Unique among parrots, kākāpō are nocturnal and exhibit a lek mating system, in which males aggregate and compete for females, increasing the risk of inbreeding (Merton et al. 1984; Powlesland et al. 1992). Once widespread across New Zealand, kākāpō populations declined rapidly following human settlement, due to hunting, forest clearance, and the subsequent introduction of mammalian predators (Bergner et al. 2016; Clout and Craig 1994; Lloyd and Powlesland 1994). All extant kākāpō are descended from a small island population isolated from the mainland of New Zealand ~10,000 years ago and a single surviving male from the mainland (Dussex et al. 2018), significantly restricting their genetic diversity and exacerbating the chances of consanguineous matings in the current managed population. As a part of an intensive management regime to promote the recovery of the once‐widespread species, RRS data and later WGS data have been generated for nearly all individuals in the current population, providing opportunities to assess the robustness of RRS approaches for studying inbreeding and to evaluate additional benefits gained by investing in WGS data. Data relating to the reproductive fitness of sequenced kākāpō are also available (Digby et al. 2023), permitting improved inbreeding‐fitness correlation approaches that incorporate genome‐derived metrics such as the ROH inbreeding coefficient (*F*_ROH_).

Recent WGS analysis of modern and historical samples from the two ancestral founding populations of kākāpō suggests that many deleterious mutations present in mainland individuals were purged from the island population (Dussex et al. 2021), likely as a result of increased inbreeding and purifying selection (Dussex et al. 2023; Kyriazis et al. 2021; Pérez‐Pereira et al. 2022; Robinson et al. 2021). The impacts of this loss of deleterious genetic variants on reproductive fitness are important to understand, given that the majority of extant kākāpō are exclusively descended from the population that underwent purging. However, evidence for inbreeding depression in kākāpō remains elusive. Previous studies using microsatellites found evidence of inbreeding depression for sperm quality and hatching success (White 2012; White et al. 2015), but in contrast, inbreeding levels estimated from RRS were not associated with the survivorship of the most and least inbred offspring (Foster et al. 2021). While these studies provided important insights into population history and genome‐wide inbreeding–fitness relationships, they did not explicitly evaluate how different sequencing approaches influence ROH‐based estimates of inbreeding, or how chromosome‐level analyses can be used to investigate the genomic architecture of inbreeding depression. Studies comparing microsatellites with tens of thousands of SNP markers reveal that genotype–phenotype associations made with smaller panels of markers may not have enough statistical power to detect the realised variation in inbreeding (Balloux et al. 2004; Hoffman et al. 2014; Slate et al. 2004), highlighting the necessity for studies to re‐analyse phenotype data with high‐resolution SNP markers.

Managing inbreeding and its fitness consequences is a central challenge in the conservation of small, isolated populations. In this study, we evaluate RRS and WGS approaches for studying correlations between inbreeding and reproductive fitness (i.e., inbreeding depression) in kākāpō using a common set of 148 individuals, representing the majority of adults alive at the time of sequencing. We compare the characteristics of ROH identified by each dataset after applying a series of optimisation approaches rarely utilised in conservation genetics. We then use a multimodel inference approach to examine potential relationships between reproductive traits of female kākāpō and individuals' levels of inbreeding (*F*_ROH_) and evaluate whether and to what extent RRS and WGS datasets produce congruent results. Additionally, using WGS we extend tests to individual chromosomes (*F*_ROH_CHR_) to accommodate the detection of potential localised effects of ROH on reproductive traits, thereby ascertaining whether the scale at which inbreeding is considered can influence the detection of inbreeding depression. Finally, we discuss the implications of these results for kākāpō management and the effectiveness of RRS and WGS approaches for studying inbreeding depression in a conservation context.

## 2 Methods

### 2.1 Study Population

The extant kākāpō population is descended from survivors of two distinct founding populations translocated during efforts to rescue the species from extinction. Between 1974 and 1977, surveys were undertaken in Fiordland on the mainland of New Zealand, and a small population of 18 individuals was discovered (Clout and Merton 1998; Powlesland et al. 2006). However, all individuals were male and only one survived to contribute genetically to the current population. Later in 1980, a breeding population was discovered on Stewart Island, 30 km south of the mainland of New Zealand, and approximately 60 individuals were relocated to offshore islands up until 1992. Genomic evidence indicates that these two populations constituted distinct lineages, with each contributing different levels of genetic diversity, inbreeding and mutational loads to the extant population (Dussex et al. 2021; Foster et al. 2021). Due to the long lifespan of kākāpō (potentially > 90 years), founders from Stewart Island are still alive and contribute to the population today, with some continuing to reproduce and others having never produced offspring despite surviving into advanced age. The combination of originating from a small insular population, infrequent breeding triggered by rimu masting, and a lek mating system in which a dominant male can father most offspring predisposes the kākāpō to inbreeding (Clout and Merton 1998; Robertson 2006). The current kākāpō population totals 237 individuals (as of December 2025) located across several predator‐free offshore islands of New Zealand.

### 2.2 DNA Extraction and Sequencing

Kākāpō samples have been continually collected and stored since intensive management of the species began, including the majority of founding individuals and their descendants. Whole blood was collected and stored in lysis buffer (Seutin et al. 1991) or absolute ethanol at −20°C until DNA extraction. Genomic DNA was isolated using a standard phenol‐chloroform extraction following Sambrook et al. (1989). A total of 148 individuals included in this study were sequenced using a whole‐genome sequencing (WGS) and a reduced‐representation sequencing (RRS) approach. RRS libraries were double‐digested with restriction enzymes PstI and MspI, and sequenced single‐ended on an Illumina HiSeq2500 (see Foster et al. 2021, for detailed description of RRS methods). The kākāpō reference genome utilised in variant calling was constructed using long‐read PacBio and Hi‐C libraries, and long‐range scaffolding using Bionano optical mapping, as a part of the Vertebrate Genome Project (VGP) and avian B10K project (GenBank assembly accession: GCF_004027225.2). All other individuals were resequenced using paired‐end whole‐genome sequencing (2 × 150 bp) on an Illumina HiSeq2500. See Dussex et al. (2021) and Guhlin et al. (2023) for extended reference genome assembly and WGS methods.

### 2.3 Variant Calling and Filtering

#### 2.3.1 Whole‐Genome Sequencing (WGS)

All sequence data was processed, filtered and analysed on the New Zealand eScience Infrastructure (NeSI) high‐performance computing facilities. Quality‐verified and trimmed reads were mapped to the reference genome using BWA *mem* v0.7.15 (Li 2013), and single nucleotide polymorphisms (SNPs) were called using BCFtools *mpileup* and *call* v1.10.2 (Li 2011a, 2011b). Further details including quality control, adaptor removal, alignment assessment, addition of read groups, marking of duplicate reads and variant calling procedures are available in the Supporting Information.

Variant filtering was performed using a combination of genotype‐level and site‐level criteria. Using BCFtools *filter*, genotype calls were first filtered to retain genotypes with a minimum depth (DP) greater than four, a quality greater than 20, heterozygous allelic balance < 0.2 and > 0.8, and with at least five reads covering the forward and reverse reference and alternate bases (DP4). Site‐level filtering was then applied to the resulting variant set. Repeat regions annotated by RepeatMasker were excluded by intersecting variant calls with a BED file obtained from NCBI using BEDtools *intersect* v.2.29.2 (Quinlan and Hall 2010), and further SNP filtering was performed in VCFtools v1.15 to remove sex chromosomes, allow up to 10% missing data per site, and only include biallelic sites (Danecek et al. 2011). Filtering for minor allele frequency (MAF) and linkage disequilibrium was not performed, as this can substantially reduce the size of the dataset and bias inbreeding estimates, particularly when considering the small sample size and population structure of kākāpō. Next, Mendelian errors were estimated using known parentage and offspring identities with PLINK v1.09 (Purcell et al. 2007), and erroneous genotypes were removed using VCFtools. Extended methods for variant processing and filtering are included in Supporting Information.

#### 2.3.2 Reduced‐Representation Sequencing (RRS)

Stacks v2.4 *process*_*radtags* was used to demultiplex the raw RRS reads and to trim barcodes (Catchen et al. 2013, 2011). Quality‐verified reads were mapped to the reference genome using BWA *mem* v0.7.15, and SNPs were called using BCFtools *mpileup* and *call* v1.10.2.

The RRS dataset was filtered similarly to WGS but with less stringency to maximise the number of SNPs retained and improve detection of ROH. Genotype‐level filtering was applied using BCFtools *filter* and *view* to include sites with a depth (DP) greater than one, quality greater than 20, heterozygous SNPs with an allelic balance < 0.2 and > 0.8, and having at least two reads covering the forward and reverse reference and alternate bases (DP4). Site‐level filtering then excluded SNPs overlapping repeat regions using BEDtools *intersect* v.2.29.2. Further SNP filtering was applied in VCFtools v1.15 including removing the sex chromosomes, allowing up to 20% missing data per site, and only retaining biallelic sites (Paris et al. 2017; Shafer et al. 2017). Finally, Mendelian errors were estimated and removed using the same procedure as for WGS. Detailed methods for quality control, adaptor trimming and extended methods for variant processing and filtering are included in Supporting Information.

### 2.4 Estimates of Inbreeding: Runs of Homozygosity (ROH)

Individual levels of inbreeding were assessed in all 148 kākāpō by identifying runs of homozygosity (ROH). The identified ROH can then be used to calculate *F*_ROH_—an inbreeding coefficient that quantifies the proportion of the genome covered by ROH. ROH were identified in WGS and RRS datasets using a sliding window approach implemented in PLINK (*‐‐homozyg*) v.109 (Purcell et al. 2007). Numerous parameters can be adjusted in PLINK that affect the detection of ROH. Therefore, initial PLINK parameters were chosen based on the parameter values used by other relevant ROH studies (see Table S1), and then a series of optimisation steps were applied to the WGS and RRS datasets to adjust parameter values based on the specific characteristics of each dataset. First, *L* (the ‘L‐parameter’), the number of homozygous SNPs per window required to form a ROH segment (PLINK parameter *‐‐homozyg‐snp*) was determined for each dataset as (Lencz et al. 2007; Meyermans et al. 2020; Purfield et al. 2012):

$$L=\frac{\log_{e}\frac{\alpha }{n_{s}n_{i}}}{\log_{e}\left(1-\mathrm{het}\right)}$$

where *α* is the false positive rate (0.05), *n*_*s*_ is the number of SNPs, *n*_*i*_ is the number of individuals genotyped, and *het* is the mean heterozygosity across all SNPs genotyped. Second, Meyermans' genome coverage parameter (Meyermans et al. 2020), expressed as a percentage, was calculated for each dataset to determine which combination of PLINK parameter values maximised genome coverage in the ROH analysis (see Supporting Information for details). Next, the overall SNP density of each dataset was evaluated to ensure that they reached minimum recommended density thresholds for ROH analysis in PLINK (Ferenčaković et al. 2013; Lavanchy and Goudet 2023). No explicit minimum ROH length threshold was imposed, as ROH length is implicitly constrained by SNP density, window‐based parameters, and the L‐parameter, allowing both short and long ROH to contribute to genome‐wide estimates of autozygosity.

Employing the PLINK ‐‐*homozyg* function, the following parameters were used to define homozygous regions (ROH) for WGS: minimum of 40 homozygous SNPs (‐‐*homozyg‐snp* 40, ‘the L‐parameter’), window size of 300 kb (*‐‐homozyg‐kb* 300), minimum SNP density of one SNP every 100 kb (*‐‐homozyg‐density* 100), maximum distance between neighbouring SNPs of 1 Mb (*‐‐homozyg‐gap* 1000), and a maximum of three heterozygous sites (*‐‐homozyg‐het* 3). In addition, each window required > 50 SNPs (*‐‐homozyg‐window‐snp* 50), contained a maximum of three heterozygous sites (*‐‐homozyg‐window‐het* 3), and was allowed no more than five missing site calls (*‐‐homozyg‐window‐missing* 5). For RRS, the same parameters were used to define homozygous regions (ROH) except for the following parameters: minimum of 37 homozygous SNPs (*‐‐homozyg‐snp* 37, ‘the L‐parameter’), minimum SNP density of one SNP every 130 kb (*‐‐homozyg‐density* 130), and the sliding window required > 25 SNPs (*‐‐homozyg‐window‐snp* 25).

The R package *detectruns* v0.9.6 (R v.4.0.3; R Core Team 2020) was used to provide summary statistics for the PLINK ROH outputs from each dataset (Biscarini et al. 2019). Next, the *F*_ROH_ inbreeding coefficient was calculated for each individual based on the ROH detected in WGS and RRS datasets:

$$F_{\mathrm{ROH}}=\frac{\sum L_{\mathrm{ROH}}}{L_{\text{Auto}}}$$

where ∑ *L*_ROH_ is the sum of the total length of an individual's ROH and *L*_Auto_ is the autosomal genome length (McQuillan et al. 2008). Additionally, *F*_ROH_ was calculated on a per chromosome basis (*F*_ROH_CHR_), and *F*_ROH_ and *F*_ROH_CHR_ values for each individual were compared between WGS and RRS datasets using Pearson's correlations computed and visualised using the R package cor.test and *ggplot2* v3.3.5 (Wickham 2016). Correlations between levels of variant missingness and the *F*_ROH_ values of each individual were also examined to confirm that *F*_ROH_ values were not affected by missing data. Finally, the distributions of ROH across chromosomes were visualised by adapting the custom *ggplot2* script provided by Stoffel et al. (2021a).

### 2.5 Inbreeding and Fitness Correlations

#### 2.5.1 Phenotypes

Individual‐level phenotypic data has been collected by the Kākāpō Recovery Team since the early 1980s as a part of routine health checks and intensive monitoring during breeding seasons. Since kākāpō are radio‐tagged and the nesting locations of female kākāpō can be located, data on a variety of reproductive traits have been collected and can be assigned to individuals. During breeding seasons, mated females lay between one and four eggs several days apart, which are monitored regularly and typically hatch within 30 days (Eason et al. 2006). However, some eggs are infertile and a large proportion of fertilised eggs suffer from early embryo mortality, with approximately 61% of eggs failing to hatch, the majority of which are fertilised but arrest early in development, highlighting this life stage as a point at which inbreeding depression may be expressed (Savage et al. 2021; White et al. 2015). Second clutches can be induced if the first clutch fails (e.g., infertile, crushed) or if eggs are removed from the nest and artificially incubated to increase population numbers when the female is in good condition. Traits representing female reproductive success examined here include clutch size (*n* = 49), the total number of eggs hatched (‘hatching success’; *n* = 49) and egg volume (per egg, *n* = 47). Clutch size and hatching success were analysed for the same set of females, whereas egg volume was analysed for a slightly smaller subset due to incomplete egg measurements for some individuals.

For each reproductive trait, the number of replicated measurements (e.g., for egg volume) and the individuals included vary due to the nature of how the data was collected. Clutch size includes the number of eggs from both first and second clutches laid by a female, and the total number of eggs hatched includes both naturally and artificially incubated eggs since, in some breeding seasons, many eggs are collected for incubation or are cross‐fostered to other females. Given that management interventions varied across years, excluding such eggs would significantly reduce sample size, and the total number of eggs hatched was therefore retained as the most consistently comparable measure of reproductive output. Egg volume was calculated from the lengths and widths of eggs by applying a constant described by Narushin (2005) for egg geometry calculations (confirmed with equation taken from Eason et al. (2006); *R* ^2^ = 0.99). Data on egg fertility was not included, as a recent study by Savage et al. (2021) found that visual inspection of kākāpō eggs by candling was not sufficiently accurate at distinguishing infertile eggs from early death embryos. Female age and generation were also considered as factors potentially influencing female reproductive traits. The age of each individual was determined from their hatch date, when known, and the age of founders was estimated by assigning a minimum age of 10 years at capture if they were confirmed to be adults when found.

Across all traits, 49 female individuals had sufficient data available for analysis, with reproductive records spanning breeding seasons from 1981 to 2019 and representing up to three generations (with lower‐numbered generations corresponding to earlier generations in models). The age of females ranged from 8 to 52 years, with a mean age of 27.8 years. Clutch size varied between 1 and 5 eggs (mean = 2.74), egg volume ranged from 14.51 to 47.76 mL (mean = 38.57 mL), and the number of eggs hatched ranged from 0 to 4 (mean = 1.06).

#### 2.5.2 Statistical Analysis

Generalised linear mixed‐effects models (GLMMs) were used to investigate the impact of inbreeding (*F*_ROH_) on female reproductive success for WGS and RRS datasets. The response variables considered in the models included clutch size, hatching success, and egg volume; the predictor variables included age, generation and inbreeding (*F*_ROH_). Individual ID and year of data collection were included as random effects to account for repeated measures across females and temporal differences in management practices. A clutch‐level random effect (clutchID) was also included, defined as a unique identifier for each female–year–clutch combination, to account for repeated measurements taken from the same clutch. Collinearity among the predictor variables was assessed by calculating the variance inflation factor (VIF) and, as a result, age was not included in egg volume models. Separate models were fitted for each reproductive trait using the *glmer* function of the R package *lme4* (Bates et al. 2015) with a Poisson error structure for hatching success and clutch size, and Gaussian for egg volume. Following Grueber et al. (2011), models were standardised using the R package *arm* (Gelman et al. 2015), and the four models with the highest AICc values (Akaike Information Criterion corrected for small sample size) were selected for model averaging in the *MuMIn* package (Barton 2009). Interpretations of models relied on the effect sizes, standard errors, and relative importance (RI) values of the final model averaging results, and were considered to be strongly supported if 95% confidence intervals do not cross zero.

To investigate chromosome‐level effects of inbreeding, multilevel Bayesian models were fitted in the *brms* package (Bürkner 2017), which uses Stan for Hamiltonian Monte Carlo sampling (Carpenter et al. 2017). Chromosome‐specific ROH values (*F*_ROH_CHR_) were included as predictors for all three reproductive traits, with Poisson error structures specified for clutch size and hatching success, and a Gaussian error structure for egg volume. Random intercepts were fitted for individual ID and chromosome. This approach accounts for the non‐independence of *F*_ROH_ values across chromosomes, since inbreeding is genome‐wide and chromosome effects represent deviations from the average genomic effect (Hewett et al. 2024). Models were run with four chains of 3000 iterations each (1500 warmup), with adapt_delta set to 0.99 to improve sampling stability. Trace plots were inspected to validate model convergence and posterior predictive checks were used to assess model fit. To evaluate whether including chromosome as a random effect improved model predictive performance, models with and without the chromosome‐level term were compared using approximate leave‐one‐out cross‐validation (LOO), implemented via the *loo* package within *brms* (Vehtari et al. 2017).

## 3 Results

Two high‐quality SNP datasets were generated from WGS and RRS data available for a common set of 148 individuals. WGS data mapped to the reference genome with a mapping rate of 99% and a mean depth of 23.31×, and RRS data mapped with a mapping rate of 95% and a mean depth of 8.14×. The initial WGS variant dataset included 2,336,466 SNPs prior to filtering and, after removing Mendelian errors, sex chromosomes, and sites with high levels of missing data, a total of 1,552,757 SNPs were retained for subsequent analyses. The initial RRS variant dataset included 66,749 SNPs prior to filtering, and after applying comparable filtering to that of the WGS dataset, a total of 19,341 SNPs were retained for analyses.

Based on the specific characteristics of each dataset, several optimisation steps were undertaken to maximise the power and accuracy of ROH identification, including calculation of the L‐parameter and evaluation of Meyerman's genome coverage parameter. The calculated L‐parameter values were 40 for WGS and 37 for RRS. After trialling several combinations of PLINK ROH parameter values, Meyerman's genome coverage parameter for the final selected values indicated that WGS and RRS had a genome coverage of 98.2% and 57.65%, respectively. The SNP density of each dataset was also evaluated, with WGS exhibiting a density of 1531.62 SNPs/Mb and RRS a density of 19.19 SNPs/Mb. Considering the thresholds recommended by Lavanchy and Goudet (2023) (simulated data), these densities are sufficient to identify ROH, particularly in empirical datasets from species with low genetic variability and a history of long‐term inbreeding, where extended homozygous regions are common.

**FIGURE 1:**
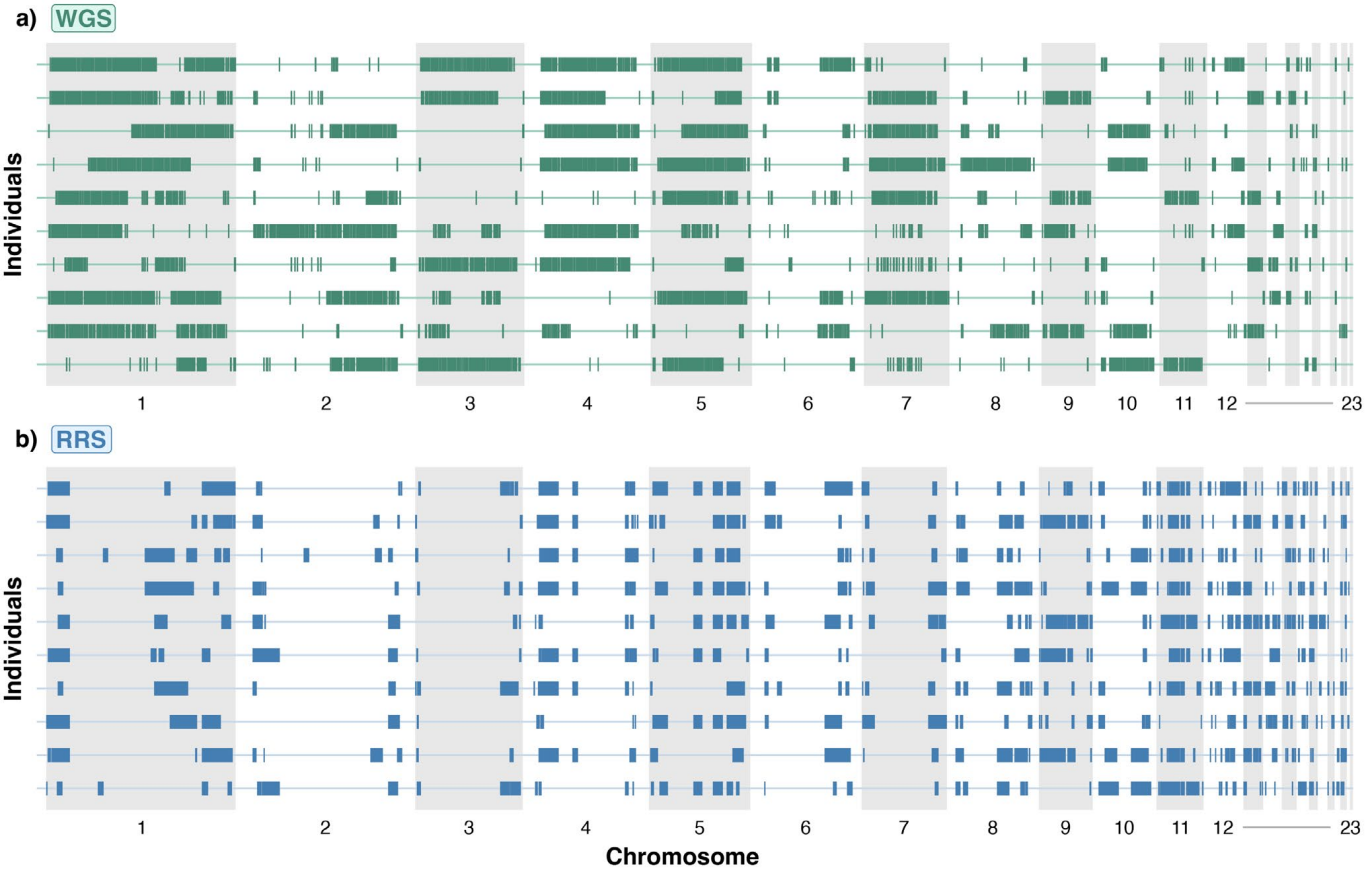
Runs of homozygosity (ROH) distributed across the genome of kākāpō. ROH detected are presented for the same 10 individuals with the highest inbreeding coefficients in (a) the whole‐genome sequencing (WGS) dataset, (b) the same 10 individuals in the reduced‐representation sequencing (RRS) dataset.

**FIGURE 2:**
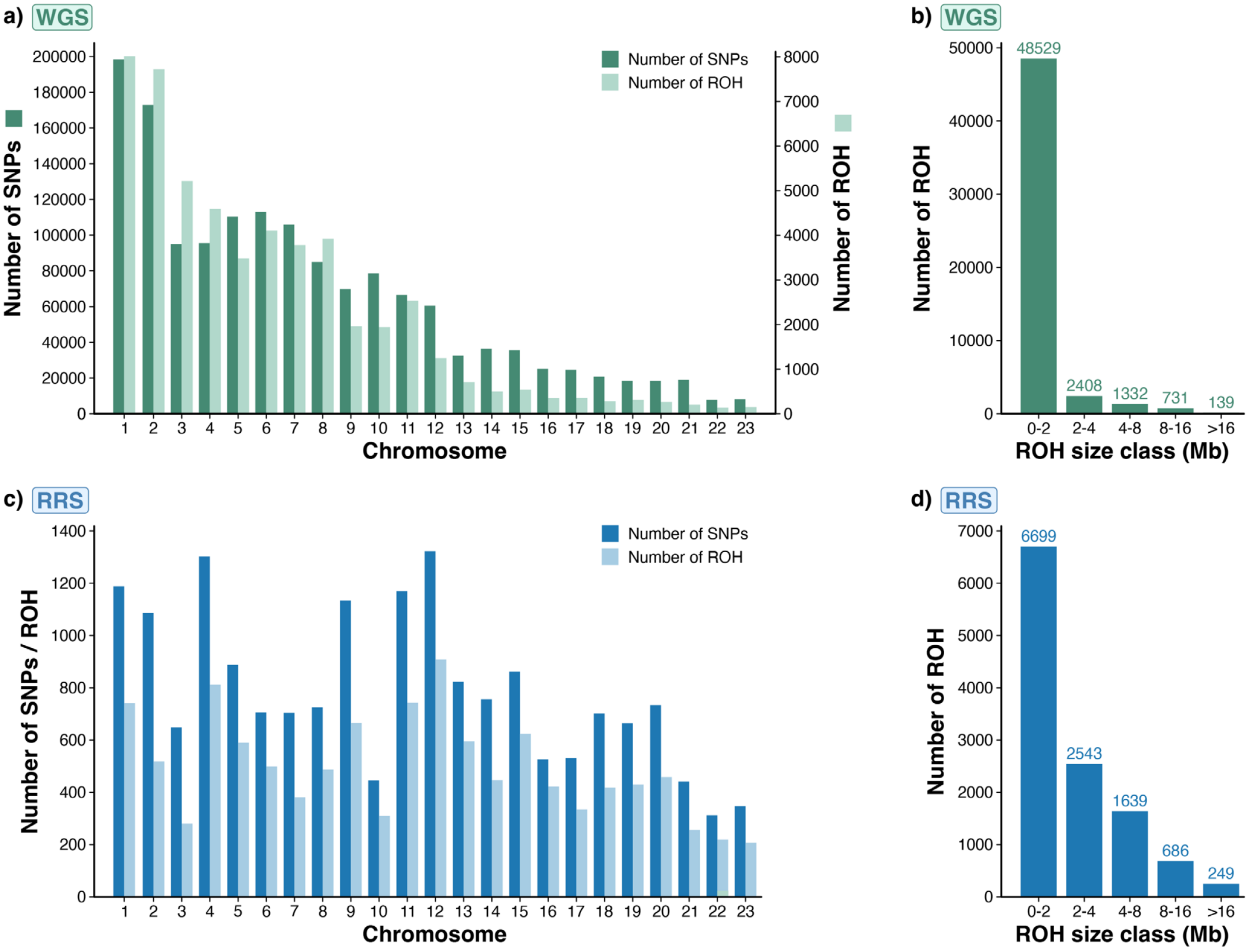
Characteristics of ROH detected among all 148 individuals. (a) Total number of SNPs and ROH detected for WGS; (b) distribution of ROH among different size classes for WGS. (c) Total number of SNPs and ROH detected for RRS; (d) distribution of ROH among different size classes for RRS. Numbers above bar plots represent the total counts for each size class.

Following optimisation, the characteristics of ROH detected across the autosomal genome were evaluated and compared between the WGS and RRS datasets. In both datasets, ROH were distributed across the genome and spanned most chromosomes except for some of the micro‐chromosomes that are too short for ROH to accumulate or be accurately detected (Figure 1). In the WGS dataset, a total of 53,139 ROH were identified across all individuals (Figure 2a), with a mean number of 366 ROH per individual and a mean total cumulative ROH length of 377 Mb. In the RRS dataset, a total of 11,816 ROH were identified (Figure 2c), with a mean number of 80 ROH per individual and a mean cumulative length of 233 Mb. ROH were further categorised into size classes, which can indicate the timing of inbreeding events (Foster et al. 2021). For WGS, 48,529 ROH were 0–2 Mb in length, 2408 were between 2 and 4 Mb, 1332 were between 4 and 8 Mb, 731 were between 8–16 Mb and 139 ROH exceeded 16 Mb (Figure 2b). For RRS, 6699 ROH were between 0–2 Mb in length, 2543 were between 2 and 4 Mb, 1639 were between 4–8 Mb, 686 were between 8 and 16 Mb and 249 ROH exceeded 16 Mb in length (Figure 2d). Despite far fewer ROH being detected in the RRS dataset (Figure 2b), both the total number and length of ROH detected per individual were correlated across the WGS and RRS datasets (number of ROH: Pearson's *r* = 0.63, *p* < 0.001, Figure S1a; length of ROH: Pearson's *r* = 0.85, *p* < 0.001, Figure S1b).

**FIGURE 3:**
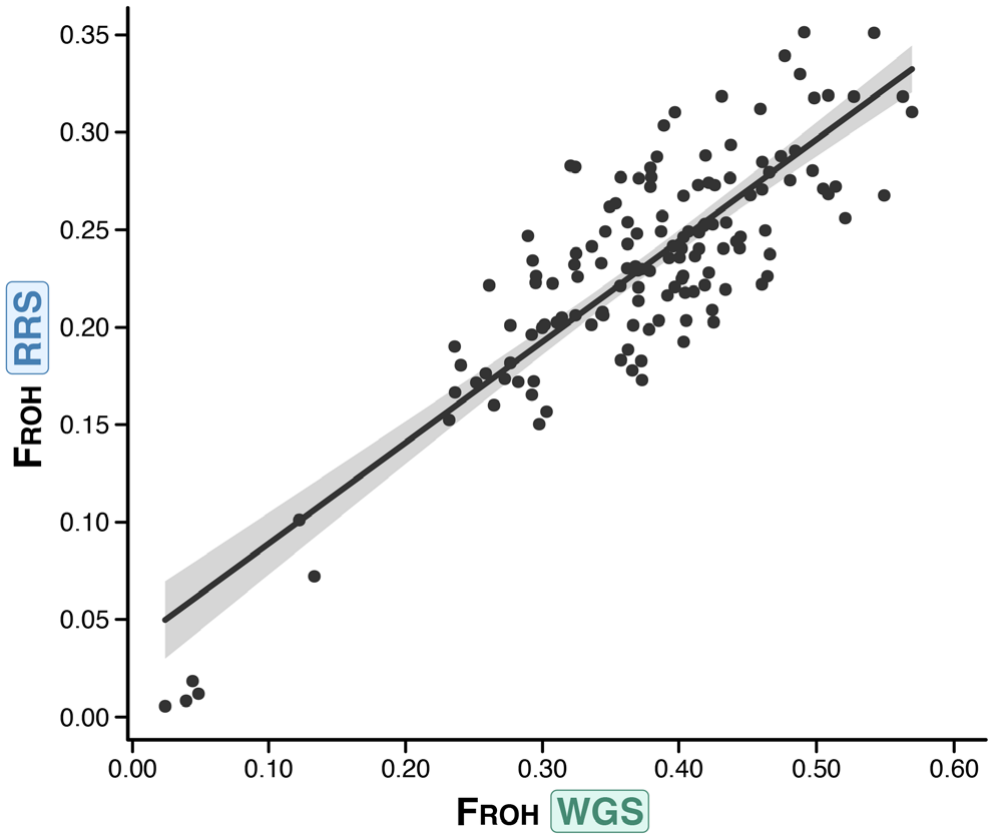
Pearson's correlation between genome‐wide inbreeding coefficients (*F*_ROH_) estimated for all individuals in the WGS and RRS datasets; *r* = 0.85, *p* < 0.05.

**FIGURE 4:**
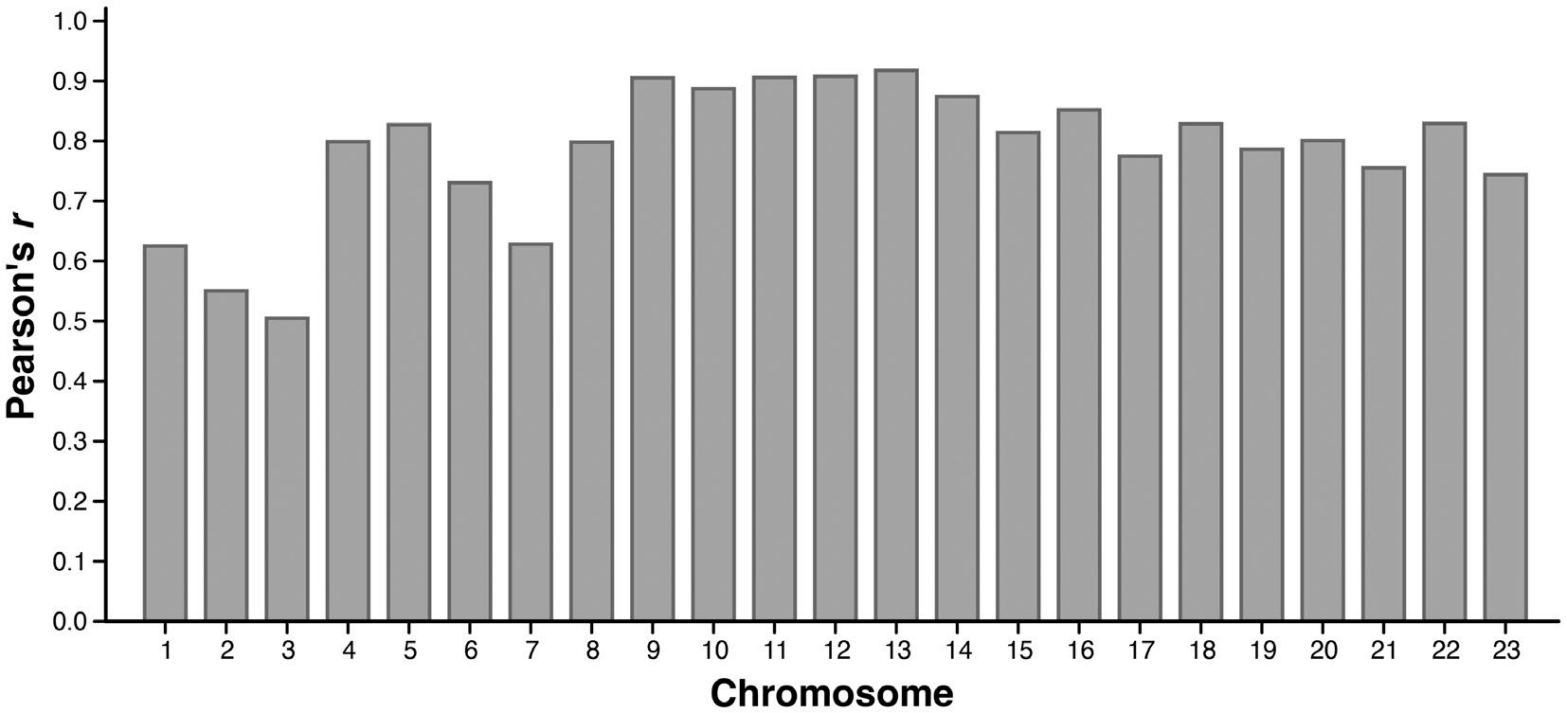
Pearson's *r* values for individual chromosomes representing correlations among chromosome‐level inbreeding coefficients (*F*_ROH_CHR_) between the WGS and RRS datasets.

Values of the inbreeding coefficient *F*_ROH_, representing the proportion of an individual's genome covered by ROH, were compared between the WGS and RRS datasets. *F*_ROH_ estimates based on WGS data ranged between 0.02 and 0.57 with a mean value of 0.37 per individual, while *F*_ROH_ estimates based on RRS data ranged between 0.01 and 0.35 with a mean value of 0.23 per individual. Correlations between individual levels of missing data and *F*_ROH_ showed no significant relationships for WGS (Pearson's *r* = −0.15, *p* = 0.078, Figure S2a) and a marginally significant relationship with RRS (Pearson's *r* = 0.17, *p* = 0.044, Figure S2b), indicating that estimates of *F*_ROH_ were unlikely to be substantially confounded by individual variation in missing data. RRS and WGS estimates of *F*_ROH_ from the same individuals were highly correlated (Pearson's *r* = 0.85, *p* < 0.001, Figure 3) indicating that RRS estimates closely reflect WGS estimates despite substantially lower SNP density. Values of the inbreeding coefficient (*F*_ROH_) were then examined per chromosome (*F*_ROH_CHR_) and compared between the datasets. Chromosomal inbreeding (*F*_ROH_CHR_) was significantly more variable than genome‐wide inbreeding (*F*_ROH_), ranging between 0.003 and 0.99 in the WGS dataset and 0.004 and 0.99 in the RRS dataset (Table S2). The chromosomes with the highest and lowest values of inbreeding varied between the two datasets, with chromosome 7 of the WGS data exhibiting the highest values of *F*_ROH_CHR_ (mean = 0.48) and chromosome 20 exhibiting the lowest (mean = 0.25), whereas chromosome 12 of the RRS dataset exhibited the highest values (mean = 0.52) and chromosome 3 the lowest (mean = 0.09). Even so, RRS and WGS estimates of *F*_ROH_CHR_ from the same individuals remained highly correlated (all *p* < 0.05) with highly congruent individual estimates of *F*_ROH_CHR_ (*r* > 0.70) produced by the two datasets across 19 of the 23 chromosomes (Figure 4).

**FIGURE 5:**
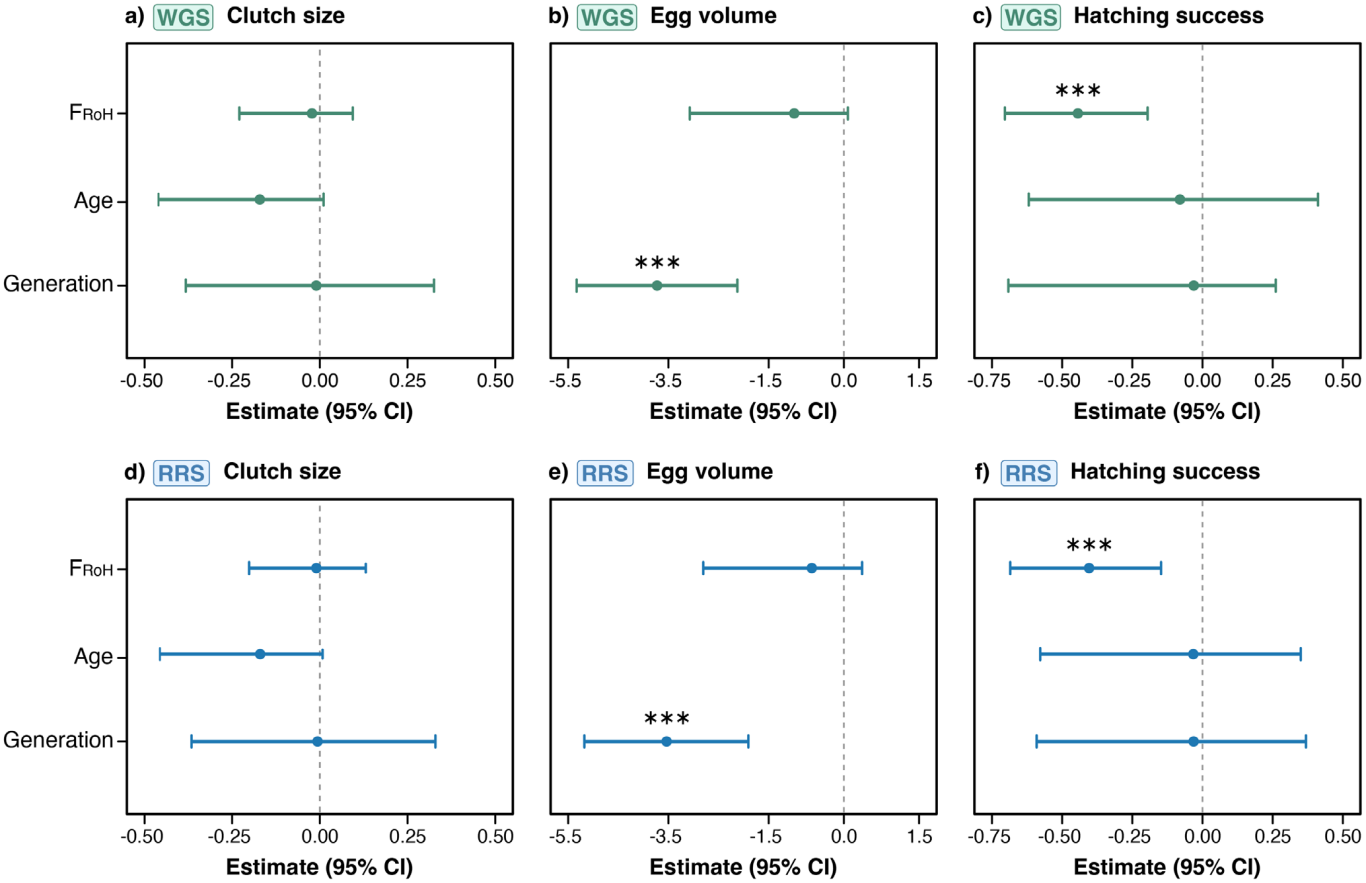
Model estimates with 95% confidence intervals (CIs) for genome‐wide inbreeding (FROH), age and generation in three components of female kākāpō reproductive success for WGS and RRS datasets. Filled circles depict the average model estimate. Components of reproductive success include clutch size (a: WGS; d: RRS), egg volume (b: WGS; e: RRS) and hatching success (c: WGS; f: RRS). Age was not included in the final models for egg volume due to high collinearity with generation.

GLMMs evaluating the effects of inbreeding (*F*_ROH_), age, and generation on reproductive success showed variable results depending on the reproductive trait being considered. However, in all instances, models based on WGS and RRS estimates of *F*_ROH_ produced similar results, suggesting a high level of agreement between the datasets (Tables 1 and 2). For models of clutch size, age received the strongest support among the predictors but was not considered to be significant, as the confidence interval marginally crossed zero (Tables 1 and 2; Figure 5a,d). Among the predictors examined for egg volume, the generation which individuals belonged to had the greatest effect and was strongly supported by the models (Tables 1 and 2; Figure 5b,e). For hatching success, inbreeding (*F*_ROH_) exhibited a negative parameter estimate with high relative importance (RI) and a confidence interval that did not cross zero, indicating strong support for reduced hatching success in more inbred individuals (Tables 1 and 2; Figure 5c,f). Global models prior to model selection are reported in Tables S3 and S4, and removing predictors to reduce model complexity did not influence the final model results (results not shown).

**TABLE 1:** Summary of GLMM parameter estimates for WGS: The effect of genome‐wide inbreeding (*F*_ROH_), age and generation on components of female kākāpō reproductive success.

Parameter	*β* (SE)	95% CI	RI
(**a** **) Clutch size**			
Intercept	0.999 (0.046)	0.908, 1.090	—
*F*_ROH_	−0.022 (0.057)	−0.229, 0.094	0.33
Age	−0.171 (0.141)	−0.459, 0.011	0.76
Generation	−0.010 (0.108)	−0.381, 0.326	0.36
**(b) Egg volume**			
Intercept	38.055 (0.478)	37.117, 38.994	—
*F*_ROH_	−0.991 (0.963)	−3.075, 0.080	0.66
Generation	**−3.726 (0.820)**	**−5.332, −2.125**	**1.00**
**(c) Hatching success**			
Intercept	0.023 (0.077)	−0.129, 0.175	—
*F*_ROH_	**−0.444 (0.138)**	**−0.704, −0.196**	**0.99**
Age	−0.031 (0.151)	−0.619, 0.411	0.30
Generation	−0.080 (0.181)	−0.692, 0.261	0.37

*Note:* Strongly supported models where 95% confidence intervals do not cross zero are indicated in bold. *β*: standardised coefficient for model predictors; SE: adjusted standard error following model averaging; RI: relative importance of each parameter to the other parameters in the final model.

Abbreviation: 95% CI: 95% confidence interval.

**TABLE 2:** Summary of GLMM parameter estimates for RRS: The effect of genome‐wide inbreeding (*F*_ROH_), age and generation on components of female kākāpō reproductive success.

Parameter	*β* (SE)	95% CI	RI
**(a) Clutch size**			
Intercept	0.999 (0.046)	0.908, 1.091	—
*F*_ROH_	−0.010 (0.047)	−0.202, 0.131	0.28
Age	−0.170 (0.140)	−0.455, 0.008	0.76
Generation	−0.006 (0.105)	−0.366, 0.330	0.35
**(b) Egg volume**			
Intercept	38.04 (0.480)	37.096, 38.984	—
*F*_ROH_	−0.640 (0.844)	−2.805, 0.364	0.52
Generation	**−3.541 (0.833)**	**−5.177, −1.905**	**1.00**
**(c) Hatching success**			
Intercept	0.018 (0.082)	−0.143, 0.179	—
*F*_ROH_	**−0.404 (0.152)**	**−0.685, −0.148**	**0.97**
Age	−0.033 (0.137)	−0.578, 0.350	0.29
Generation	−0.032 (0.139)	−0.591, 0.369	0.29

*Note:* Strongly supported models where 95% confidence intervals do not cross zero are indicated in bold. *β*: standardised coefficient for model predictors; SE: adjusted standard error following model averaging; RI: relative importance of each parameter to the other parameters in the final model.

Abbreviation: 95% CI: 95% confidence interval.

To evaluate whether inbreeding depression is driven by localised genomic effects, we examined chromosome‐specific inbreeding coefficients (*F*_ROH_CHR_) for each reproductive trait using Bayesian multi‐membership mixed models. Across all three traits, fixed effects of chromosome‐specific inbreeding were very small (posterior means approximately −0.01 to 0.00), with wide credible intervals spanning zero (Table S5), indicating a lack of strong evidence for individual chromosomes influencing reproductive output beyond the genome‐wide signal. Leave‐one‐out cross‐validation further supported the absence of chromosome‐level effects, with models excluding chromosome as a random effect providing equal or slightly improved predictive performance for hatching success (*Δelpd_loo* = −0.7), clutch size (*Δelpd_loo* ≈0) and egg volume (*Δelpd_loo* = −1.6). Variance attributed to chromosomes was minimal in all models, while individual‐level variance remained substantial, suggesting that differences among females rather than specific chromosomes accounted for most explained variation in reproductive traits. Taken together, these results indicate that inbreeding depression in kākāpō is unlikely to be driven by loci of large effect restricted to particular chromosomes. Instead, the pattern is more consistent with small dispersed effects of inbreeding distributed across the genome, supporting a polygenic architecture for reproductive traits in this species.

## 4 Discussion

In this study, whole‐genome sequencing (WGS) and reduced‐representation sequencing (RRS) were used to examine inbreeding and its repercussions on female reproductive success in kākāpō. Both datasets contained the same individuals, representing the majority of living kākāpō at the time of sequencing in 2016, and were processed using consistent approaches to enable equitable comparisons between the two sequencing approaches. SNPs and ROH were distributed across the genome of both datasets with predominantly short ROH segments, indicative of background relatedness or inbreeding arising from distant common ancestry (Figures 1 and 2a,c) (Kirin et al. 2010). A smaller proportion of long ROH (8–16 and > 16 Mb) were detected across both datasets, indicative of significant recent inbreeding (i.e., coalescence, Figure 2b,d) (Martin et al. 2023; Ochoa and Gibbs 2021). Dussex et al. (2021) link these long ROH segments in kākāpō to recent inbreeding, with substantially more long ROH found in the Stewart Island founding population where a large number of consanguineous matings have occurred. In contrast, a small number of individuals exhibited substantially lower ROH, a pattern consistent with known population structure and mainland ancestry in kākāpō. Long ROH were occasionally also concentrated towards the ends of some chromosomes (Figure 1), which may be explained by the lower rates of recombination in these regions (e.g., Stanhope et al. 2023).

At a preliminary level, concordance between the RRS and WGS datasets is not immediately apparent given that the number of ROH segments and their delineation varies between the datasets for the same individuals (Figure 1). For instance, RRS frequently failed to complete long ROH segments that are present in individuals in the WGS dataset, and occasionally detected short ROH that are absent from the WGS (Figure 1a,b). This outcome is expected since the RRS captures only a subset of the SNPs present in the WGS dataset (Figure 2a,c), with over a 40% difference in genomic coverage based on Meyerman's genome coverage parameter (Meyermans et al. 2020). Yet, upon conversion of the raw ROH data into the inbreeding coefficient *F*_ROH_, the concordance between the two datasets becomes apparent (Figure 3). Importantly, this concordance preserves relative differences among individuals relevant to inbreeding depression, while absolute estimates of ROH and F_ROH_ are systematically lower in RRS than in WGS, limiting population‐level inference. Consistent with this, RRS and WGS estimates of *F*_ROH_ were strongly correlated across individuals and chromosomes (Figure 4), although variation in correlation strength likely reflects uneven chromosomal SNP coverage in RRS. Together, these findings add to a growing body of evidence that ROH analyses can be effectively performed using high‐quality RRS datasets when SNP density requirements are met, particularly in highly homozygous species such as kākāpō (Duntsch et al. 2021; Lavanchy and Goudet 2023; Wright et al. 2020). Other approaches to improving genome‐wide SNP coverage, such as restriction enzyme optimisation, double digests and increasing sequencing capacity, are expected to enhance the utility of RRS datasets for ROH analyses (Catchen et al. 2017). Although WGS remains superior for characterising the full genomic landscape of autozygosity, RRS data can reliably capture overall levels of inbreeding and relative differences among individuals in highly inbred species such as kākāpō.

After obtaining robust estimates of genome‐wide inbreeding (*F*_ROH_) and chromosomal inbreeding (*F*_ROH_CHR_), potential correlations between inbreeding levels and female reproductive traits were evaluated. Models examining genome‐wide inbreeding (*F*_ROH_) also considered the age and generation of individuals as additional factors, which were found to have consistent effects on reproductive traits regardless of whether *F*_ROH_ was based on the WGS or the RRS dataset (Tables 1 and 2). Genome‐wide inbreeding (*F*_ROH_) was found to be the most strongly supported predictor of hatching success and had a significant negative effect on this trait (Figure 5c,f); that is, more inbred females containing a higher proportion of ROH across their genome had lower hatching success. Correlations between levels of inbreeding in kākāpō and hatching success were also found by White et al. (2015) based on microsatellite heterozygosity. Both WGS and RRS data from an expanded set of individuals in the current study provide strong corroborating evidence for inbreeding depression in this trait. Clutch size, however, was not significantly affected by inbreeding as in White et al. (2015). Rather, the strongest predictor of clutch size in the current study was age (Figure 5a,d), suggesting that reductions in the clutch size of female kākāpō may primarily relate to the physiological deterioration of older individuals, as was also recently found in the houbara bustard (Rabier et al. 2021). In contrast, egg volume had a significant negative relationship with generation (Figure 5b,e), suggesting that older females produce larger eggs than females from the more recent generations, conceivably due to factors such as body condition, prior breeding experience and foraging ability.

When inbreeding was examined at the chromosome level (*F*_ROH_CHR_), we found no strong evidence that reproductive traits were associated with the ROH content of specific chromosomes once genome‐wide inbreeding was accounted for. These findings suggest that inbreeding depression in kākāpō is unlikely to be driven by a small number of genomic regions of large effect. Instead, the pattern is more consistent with many loci of small effect contributing collectively to reduced reproductive performance, supporting a predominantly polygenic basis for inbreeding depression in this species. This aligns with recent work in wild systems (e.g., Hewett et al. 2024), reinforcing the idea that in historically bottlenecked populations, fitness effects may be distributed widely across the genome rather than concentrated in specific regions. This pattern may also reflect the legacy of historical purging in kākāpō. Dussex et al. (2021) reported reduced genome‐wide mutational load relative to historical specimens, suggesting that some strongly deleterious alleles may have been removed during prolonged periods of small population size. However, while purging can diminish the influence of large‐effect mutations, it is unlikely to eliminate the cumulative impact of numerous mildly deleterious alleles that continue to contribute to inbreeding depression (Dussex et al. 2023). Our results highlight the importance of managing genome‐wide diversity in conservation breeding programmes. From an applied perspective, strategies that minimise overall relatedness between breeding pairs are likely to be more effective than approaches that attempt to target or avoid specific chromosomal regions.

An understanding of how inbreeding impacts reproductive success is a valuable asset to a conservation programme with potential impacts on management decisions. In this study, the detection of strongly supported correlations between individual levels of inbreeding (*F*_ROH_) and hatching success has the potential to shape such decisions in kākāpō. Planned matings and artificial insemination are currently used to minimise inbreeding in kākāpō, wherein individuals are paired together based on their scores in a genetic relatedness matrix (Bergner et al. 2014). Given the importance of hatching success as a key determinant of overall breeding success, a revised breeding decisions framework may be warranted in which both levels of inbreeding and relatedness are considered when choosing individual pairings. Further, the finding of a significant relationship between generation and egg volume carries with it potential management implications. Further research is needed to disentangle potential factors such as individual nutrition, given that females from the founding generation have spent a significantly larger portion of their lives without artificial supplementary feeding (Powlesland and Lloyd 1994). A more comprehensive understanding of factors influencing female reproductive success may be gained by also considering the interactions between male and female kākāpō during mating, such as sperm quality (White et al., unpublished) and the number of individuals a female mated with before producing eggs, as multiple copulations have been linked to increased egg fertility in kākāpō (Digby et al. 2023). However, although a large variety of phenotypic data has been collected in kākāpō since the early 1980s, the feasibility of an expanded analysis including additional traits is currently restricted by small sample sizes and issues surrounding the consistency and continuity of data collection. For example, kākāpō eggs that were considered infertile for the majority of their management since the 1970s were later found to contain a large proportion of early death embryos (Savage et al. 2021), suggesting that other factors such as congenital defects (e.g., dwarfism) and inbreeding depression acting on early survival may be more of a limitation than adult fertility itself (Assersohn and Marshall 2021; Keller and Waller 2002). Short‐term proxies of reproductive fitness within an individual's lifetime may also reveal weaker signatures of inbreeding depression than the lifetime breeding success of multiple generations, as was recently found in helmeted honeyeaters (Harrisson et al. 2019). Therefore, the continuation of long‐term phenotypic data collection in kākāpō remains important towards gaining a more comprehensive understanding of inbreeding depression (Bérénos et al. 2016; Stoffel et al. 2021b), and its potential alleviation over time with management interventions.

While WGS enables a deeper evaluation of the genomic architecture of inbreeding depression, this study highlights the potential of using high‐quality RRS datasets for ROH analysis in a conservation context. The RRS dataset used in this study provided robust estimates of inbreeding and could be effectively used to evaluate associations with reproductive success in kākāpō, closely agreeing with individual inbreeding estimates produced by the WGS dataset and leading to the same interpretations regarding female reproductive traits impacted by inbreeding depression. Model‐based ROH callers (e.g., RZooRoH) may offer advantages for reduced‐representation datasets by accommodating ungenotyped regions and represent a valuable direction for future work. In the present study, the use of a consistent PLINK‐based framework allowed direct comparison between WGS and RRS, providing insights relevant to conservation management and resource allocation, particularly where generating WGS data across many individuals remains prohibitively expensive. It should be noted that analysis of ROH using RRS datasets still requires a chromosome‐level or scaffolded reference genome to provide genomic coordinates of SNPs. However, obtaining a high‐quality reference genome for a single individual is becoming increasingly affordable (Formenti et al. 2022) or could be substituted with the reference genome of a closely related species (Galla et al. 2019). Although WGS still offers clear benefits when examining gene function and the genetic basis of traits, these more functional assessments are rarely a priority in conservation programmes. RRS datasets are well‐equipped to assess inbreeding and other aspects of ‘genetic health’ in endangered populations, which remain areas of high priority in conservation genetics. Crucially, RRS also allows conservation programmes with limited resources to maximise the number of individuals included in these assessments because of the significantly lower cost per individual compared to WGS.

## Author Contributions

Study conceptualisation, Y.F., B.C.R., J.C.M. Generation of data, F.R., K.D., R.B., J.J. Data processing and analysis, Y.F. Data interpretation and support, Y.F., B.J.F., L.D., N.D., S.G., A.S., T.A. Y.F. wrote the main body of the manuscript, while all authors contributed to improving the manuscript.

## Funding

Funding was provided by the Royal Society of New Zealand's Marsden Fund project ‘Resolving the genomic architecture of hatching failure to improve conservation of endangered birds’ (Contract ID UOO1817). This project (WGS and reference genome assembly) was supported by the Genetic Rescue Foundation, University of Otago, and the Rockefeller Vertebrate Genomes (VGP) lab. Y.F. was supported by a University of Otago Doctoral Scholarship. New Zealand's national facilities are provided by NeSI and funded jointly by NeSI's collaborator institutions and through the Ministry of Business, Innovation and Employment (MBIE) research infrastructure programme. This project (RRS) was also supported by the MBIE via its funding of the ‘Genomics for Production & Security in a Biological Economy’ programme (Contract ID C10X1306).

## Conflicts of Interest

The authors declare no conflicts of interest.

## Supporting information


**Data S1:** mec70252‐sup‐0001‐Supinfo.docx.

## Data Availability

The kākāpo genome assembly is available under BioProject ID PRJNA489135, NCBI assembly GCF_004027225.2. Access to kākāpo genome sequencing and phenotype data is publicly available but managed by a committee comprising representatives from the New Zealand Department of Conservation (DOC) and Te Rūnanga o Ngāi Tahu, who retain Kaitiakitanga (i.e., governance and stewardship) over the data in accordance with the principle of Indigenous data sovereignty. To request access, users must submit an application via the Aotearoa Genomics Data Repository (https://data.agdr.org.nz).
