# Supplementary material for: Genomic Architecture of Inbreeding Depression Associated With Hatching Failure in an Endangered Parrot
Source: Mol Ecol. 2026 Jan 21;35(2):e70252. doi: 10.1111/mec.70252 (PMC12820920; doi:10.1111/mec.70252)
Supplement: Supplementary file 1 — Data S1: mec70252‐sup‐0001‐Supinfo.docx. [file MEC-35-e70252-s001.docx]

**Supplementary Material**

**Genomic architecture of inbreeding depression associated with hatching failure in an endangered parrot**

Yasmin Foster^1^, Stefanie Grosser^1^, Brodie J. Foster^1^, Nicolas Dussex^2^, Aimee Stubbs^1^, Ludovic Dutoit^1^, Theo Atkinson^1^, Fiona Robertson^1^, Ken G. Dodds^3^, Rudiger Brauning^3^, Jeanne M. E. Jacobs^3^, John C. McEwan^3^ & Bruce C. Robertson^1^

1 Department of Zoology, University of Otago, PO Box 56, Dunedin 9054, New Zealand

2 Department of Population Analysis and Monitoring, Swedish Museum of Natural History, Stockholm SE-106 91, Sweden

3 AgResearch, Private Bag 50034, Mosgiel 9053, New Zealand

**Supplementary Methods**

***Study population***

The current kākāpō population totals 242 individuals (as of May 2025) located across several predator-free offshore islands of New Zealand: Whenua Hou (Codfish Island), Te Hauturu-o-Toi (Little Barrier Island), Te Kākahu-o-Tamatea (Chalky Island), and Anchor Island. There are also a small number of males housed on the mainland at Maungatautari (Sanctuary Mountain), a pest-proof fenced ecological area, as part of a trial. Most individuals have been repeatedly transferred between these islands as a part of management strategies to improve mate pairings, and therefore the location of individuals is not considered as a factor in this study. Kākāpō are managed by the Kākāpō Recovery Team, New Zealand Department of Conservation, with capture, handling, and sample collection performed in accordance with ethical requirements approved by both Ngāi Tahu and the Department of Conservation.

***Variant calling & filtering***

*Whole-genome sequencing (WGS)*

Paired-end WGS reads were trimmed using Trimgalore v0.6.4 (Krueger, 2015) to remove Illumina adapters and discard reads less than 40 bp long or with a quality of less than 20. FastQC v0.11.9 and MultiQC v1.7 were used to assess the quality of the trimmed reads (Andrews et al., 2012; Ewels et al., 2016) and the quality-verified reads were then mapped to the reference genome using BWA mem v0.7.15 (Li, 2013). After alignment, Samtools view, sort and flagstat v1.8 were used to convert file formats, coordinate-sort aligned reads, and assess alignment statistics (Li & Durbin, 2009). Picard v2.1.0 was used to add read groups to incorporate sample identity, library, and lane information into alignment files (Broad Institute, 2019). Samtools v1.8 was then used to merge multiple alignment files into a consensus alignment for each sample, and duplicate reads were marked with Picard v2.1.0. WGS variants (single nucleotide polymorphisms, SNPs) were called using BCFtools mpileup and call v1.10.2; deploying base alignment quality (BAQ) to remove false SNPs due to misalignment around indels, and removing SNPs with a mapping and base quality less than 20 (Li, 2011a, 2011b). Output files were indexed, sorted, and variant statistics were assessed with BCFtools index, sort, and stats v1.10.2. Utilising the variant statistic outputs, transition/transversion (Ts/Tv) ratios were calculated (under an expected value of approximately 2.0; Wang et al., 2015) to confirm the quality of SNP calls.

For the initial filtering of individual SNP calls, BCFtools filter and view were used to include sites with a depth (DP) greater than four, a quality greater than 20, and with at least five reads covering the forward and reverse reference and alternate bases (DP4). Heterozygous SNPs with an allelic balance <0.2 and >0.8 were also filtered out to avoid biases arising from sequencing, contamination, or mapping errors (Muyas et al., 2019). BCFtools merge was then used to merge all individual variant files into a consensus file containing all SNPs that fulfilled the initial filtering thresholds. Next, BCFtools index and view were used to index the consensus file and convert the BCF file format to VCF, which was re-indexed with Tabix (Li, 2011c). Repeat regions were then masked in BEDtools intersect v.2.29.2 (Quinlan & Hall, 2010) and further SNP filtering was performed in VCFtools v1.15 to remove the Z and W sex chromosomes, to allow up to 10% missing data, and to only include biallelic sites (Danecek et al., 2011). Next, Mendelian errors were estimated using known parentage and offspring identities with PLINK v1.09 (Purcell et al., 2007) using a custom script adapted from Humble et al. (2018), and were subsequently removed using VCFtools. A principal component analysis was performed in PLINK and, as a final quality check of the WGS data, scores of the first two principal components were visualised to confirm that the filtered dataset conformed to the known population structure of the species.

*Reduced-representation sequencing (RRS)*

For pre-processing of raw reads, Illumina adaptors, reads shorter than 20 bp long, and low-quality bases (Q<20) were removed with Trimgalore v0.6.4, and sequence quality was assessed with FastQC v0.11.9 and MultiQC v1.7. Quality-verified reads were mapped to the reference genome using BWA mem v0.7.15. After alignment, Samtools view, sort and flagstat v1.8 were used to convert files formats, sort aligned reads, and to print statistics to assess alignment mapping rate. After SNPS were called with BCFtools mpileup and call v1.10.2, output files were indexed, sorted, and variant statistics were assessed with BCFtools index, sort, and stats v1.10.2, and transition/transversion (Ts/Tv) ratios were extracted to confirm the quality of SNP calls. After initial filtering, variant files belonging to individual kākāpō were merged into a consensus file containing all SNPs with BCFtools merge. BCFtools index and view were then used to index the consensus file and perform file conversions (BCF to VCF), which was re-indexed with Tabix before repeat masking and further filtering with VCFtools.

***Estimates of inbreeding: runs of homozygosity (ROH)***

Individual levels of inbreeding were characterised in all 148 kākāpō by identifying runs of homozygosity (ROH). ROH are long tracts of homozygosity across the genome that are indicative of both distant and recent inbreeding events (Ceballos et al., 2018; Kardos et al., 2016). They serve as an absolute measure of autozygosity, reflecting the realised proportion of the genome that is identical by descent (McQuillan et al., 2008). ROH identified by this analysis can be studied directly to understand ROH length characteristics and distributional patterns, or can be used to calculate F_ROH_, an inbreeding coefficient representing the proportion of an individual’s genome covered by ROH.

We improved our ROH analysis using several optimisation steps. Meyermans’ genome coverage parameter (Meyermans et al., 2020), expressed as a percentage, was calculated for each dataset to determine which combination of PLINK parameter values maximises the coverage of the genome by the ROH analysis. To achieve this, WGS and RRS datasets were transformed into simulated datasets wherein all SNPs were made homozygous, and PLINK parameters associated with SNP density, SNP interval size, and window size were adjusted until the combination of PLINK parameter values was found that maximised genome coverage for each dataset following Gorssen et al., (2021). Additionally, special consideration was given to the window size parameter of PLINK, due to the history of long-term inbreeding in kākāpō (Foster et al., 2021) and the generally smaller avian chromosome size compared to that of humans, for whom ROH methods were developed (Machado et al., 2022). A window size of 300 kb was selected for both datasets to increase the sensitivity of detection of short ROH while still minimising the detection of false positives arising from linkage disequilibrium.

The R package *detectruns* v0.9.6 (R v.4.0.3; (R Core Team, 2020)) was used to provide summary statistics for the PLINK ROH outputs from each dataset (Biscarini et al., 2019), including the total number of ROH detected across the genome, the number of ROH detected per chromosome, the number and length of ROH detected per individual, and number and average length of ROH fragments within different size classes (0-2, 2-4, 4-8, 8-16, >16 Mb).

## Supplementary figures & tables


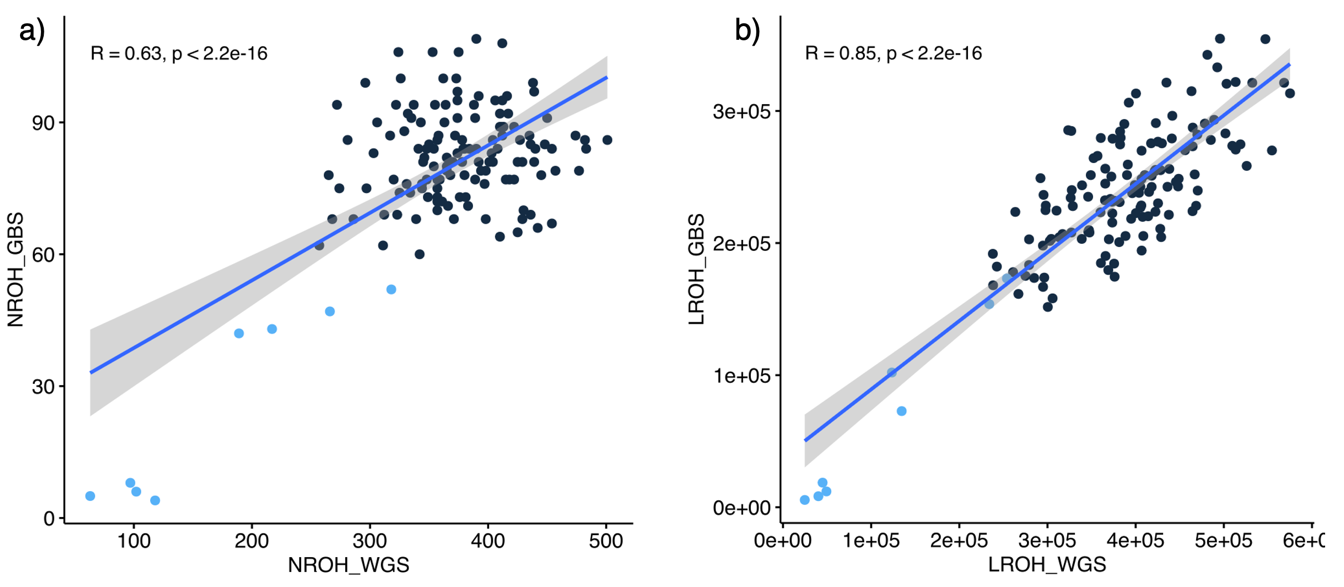


**Supplementary Figure 1.** (a) Pearson’s correlation between the number of ROH (N_ROH_) identified between WGS and RRS datasets; (b) Pearson’s correlation between the length of ROH (L_ROH_) identified between WGS and RRS datasets. Blue dots represent mainland descendants, and black dots represent Stewart Island descendants.


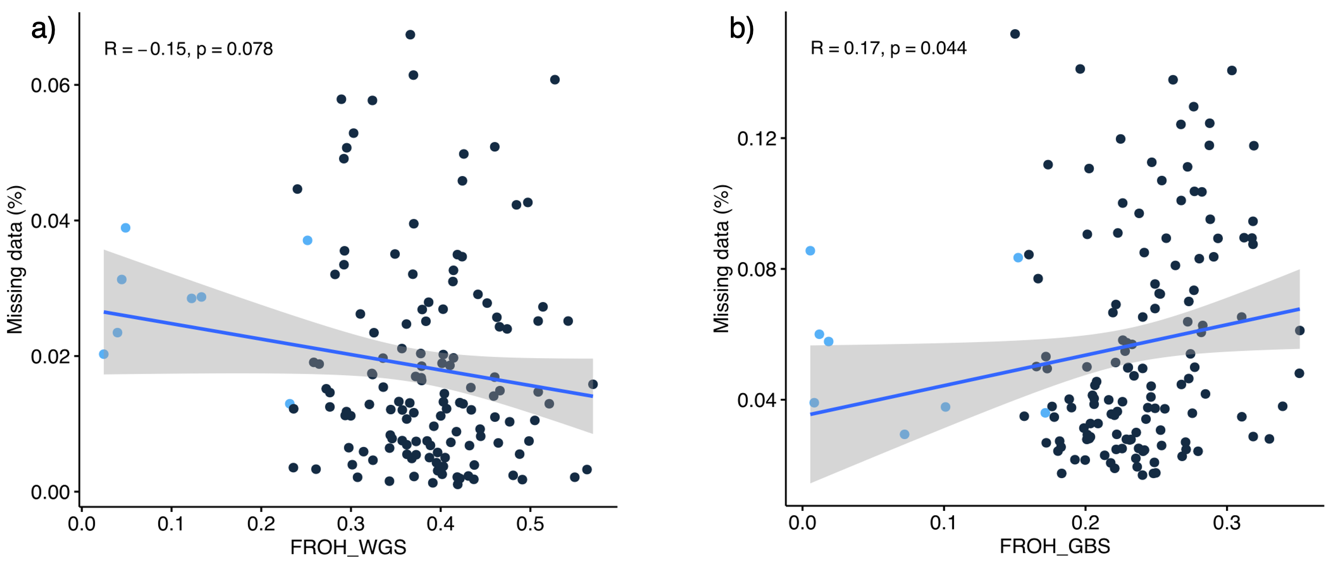


**Supplementary Figure 2.** Pearson’s correlation genome-wide inbreeding (F_ROH_) and missing data for (a) WGS and (b) RRS. Blue dots represent mainland descendants, and black dots represent Stewart Island descendants.

**Supplementary Table 1.** PLINK parameters used for ROH analysis in this study compared with 11 other studies.

| PLINK parameter | This study | 1 | 2 | 3 | 4 | 5 | 6 | 7 | 8 | 9 | 10 | 11 |
| --- | --- | --- | --- | --- | --- | --- | --- | --- | --- | --- | --- | --- |
| homozyg-snp (*L*) | 40 | 25 | 50 | 100 | – | 100 | 100 | 41 | 100 | 50 | 50 | 65  80 |
| homozyg-kb | 300 | 100 | 1200 | 1000 | – | 500 | 100 | 500 | – | 300 | – | 0 |
| homozyg-gap | 1000 | 1000 | 300 | 100 | – | 100 | 1000 | 1000 | – | 1000 | 500 | 5000 |
| homozyg-density | 100 | 50 | 200 | 50 | – | 10 | 50 | 5000 | 50 | 50 | 50 | 5000 |
| homozyg-window-snp | 50 | 100 | 50 | 50 | 200 |  | 100  200 | 41 | 50 | 50 | 100 | 65  80 |
| homozyg-window-missing | 5 | – | 2 | 5 | 50 | 5 | – | 0 | – | 5 | – | 5 |
| homozyg-het | 3 | 750 | 2 |  |  |  | 3 |  | – |  | 0 |  |
| homozyg-window-het | 3 | 1 | 2 | 1 | 3 | 2 | – | 0 | – | 1  5 | – | 0  1 |
| homozyg-window-threshold | 0.05 | 0.05 | – | – | – | – | 0.05 | 0.05 | – | 0.05 |  | 0.05 |

^1^ Dussex et al., 2021; ^2^ Stoffel et al., 2021a; ^3^ Robinson et al., 2021; ^4^ Robinson et al., 2019; ^5^ Grossen et al., 2020; ^6^ Hasselgren et al., 2021; ^7^ Sams & Boyko, 2019; ^8^ Caballero et al., 2021; ^9^ Ceballos et al., 2018a; ^10^ Alemu et al., 2021; ^11^ Howrigan et al., 2011.

^1^ Dussex21; ^2^ Stoffen21; ^3^ Robison21; ^4^ Robinson19; ^5^ Grossen20; ^6^ Hasselgren21; ^7^ Sams19; ^8^ Caballero20; ^9^ Caballos18; ^10^ Alemu21; ^11^ Howrigan11.

**Supplementary Table 2.** Minimum, maximum, and mean values of F_ROH_CHR_ across chromosomes based on (a) WGS and (b) RRS.

| **a) F_ROH_CHR_ (WGS)** | | | |  | **b) F_ROH_CHR_ (RRS)** | | | |
| --- | --- | --- | --- | --- | --- | --- | --- | --- |
| Chr. | Min | Max | Mean |  | Chr. | Min | Max | Mean |
| 1 | 0.011 | 0.885 | 0.371 |  | 1 | 0.011 | 0.524 | 0.203 |
| 2 | 0.003 | 0.977 | 0.382 |  | 2 | 0.004 | 0.277 | 0.107 |
| 3 | 0.014 | 0.981 | 0.395 |  | 3 | 0.007 | 0.255 | 0.086 |
| 4 | 0.004 | 0.979 | 0.402 |  | 4 | 0.007 | 0.462 | 0.185 |
| 5 | 0.008 | 0.947 | 0.365 |  | 5 | 0.007 | 0.604 | 0.252 |
| 6 | 0.004 | 0.929 | 0.348 |  | 6 | 0.014 | 0.490 | 0.202 |
| 7 | 0.040 | 0.990 | 0.476 |  | 7 | 0.005 | 0.369 | 0.159 |
| 8 | 0.017 | 0.955 | 0.385 |  | 8 | 0.012 | 0.749 | 0.287 |
| 9 | 0.007 | 0.943 | 0.329 |  | 9 | 0.026 | 0.924 | 0.358 |
| 10 | 0.017 | 0.992 | 0.421 |  | 10 | 0.023 | 0.768 | 0.334 |
| 11 | 0.017 | 0.884 | 0.427 |  | 11 | 0.016 | 0.890 | 0.480 |
| 12 | 0.032 | 0.910 | 0.367 |  | 12 | 0.070 | 0.947 | 0.521 |
| 13 | 0.018 | 0.898 | 0.317 |  | 13 | 0.023 | 0.912 | 0.458 |
| 14 | 0.029 | 0.916 | 0.275 |  | 14 | 0.027 | 0.976 | 0.363 |
| 15 | 0.025 | 0.740 | 0.293 |  | 15 | 0.128 | 0.820 | 0.485 |
| 16 | 0.036 | 0.996 | 0.317 |  | 16 | 0.043 | 0.999 | 0.474 |
| 17 | 0.043 | 0.868 | 0.288 |  | 17 | 0.041 | 0.956 | 0.451 |
| 18 | 0.047 | 0.924 | 0.262 |  | 18 | 0.051 | 0.956 | 0.398 |
| 19 | 0.053 | 0.752 | 0.292 |  | 19 | 0.068 | 0.925 | 0.475 |
| 20 | 0.054 | 0.795 | 0.251 |  | 20 | 0.061 | 0.906 | 0.422 |
| 21 | 0.056 | 0.911 | 0.300 |  | 21 | 0.057 | 1.000 | 0.475 |
| 22 | 0.104 | 0.772 | 0.298 |  | 22 | 0.104 | 0.999 | 0.403 |
| 23 | 0.108 | 0.717 | 0.296 |  | 23 | 0.120 | 0.940 | 0.414 |

**Supplementary Table 3.** Global model components of predictors of female kākāpō reproductive success prior to model averaging for WGS, ranked in order of their AICc support. Models included individual ID and year as random effects.

| **a) Clutch size** | | | |
| --- | --- | --- | --- |
| Parameter | AICc | △AICc | AICc weight |
| Age | 671.20 | 0.00 | 0.35 |
| Age + F_ROH_ | 672.70 | 1.44 | 0.17 |
| Age + generation | 672.90 | 1.70 | 0.15 |
| Generation | 674.10 | 2.84 | 0.09 |
| Age + F_ROH_ + generation | 674.10 | 2.92 | 0.08 |
| Null | 674.20 | 2.95 | 0.08 |
| F_ROH_ | 675.70 | 4.52 | 0.04 |
| F_ROH_ + generation | 675.80 | 4.57 | 0.04 |
|  | | | |
| **b) Egg volume** | | | |
| Parameter | AICc | △AICc | AICc weight |
| F_ROH_ + generation | 2245.98 | 0.00 | 0.66 |
| Generation | 2247.32 | 1.34 | 0.34 |
| Null | 2261.43 | 15.45 | 0.00 |
| F_ROH_ | 2261.81 | 15.83 | 0.00 |
|  | | | |
| **c) Hatching success** | | | |
| Parameter | AICc | △AICc | AICc weight |
| F_ROH_ | 606.81 | 0.00 | 0.45 |
| F_ROH_ + generation | 608.12 | 1.31 | 0.24 |
| F_ROH_ + age | 608.86 | 2.05 | 0.16 |
| F_ROH_ + generation + age | 609.25 | 2.44 | 0.13 |
| Null | 615.30 | 8.49 | 0.01 |
| Generation | 616.97 | 10.16 | 0.00 |
| Age | 617.28 | 10.47 | 0.00 |
| Generation + age | 618.88 | 12.07 | 0.00 |

△AICc reflects the differences among selected models.

**Supplementary Table 4.** Global model components of predictors of female kākāpō reproductive success prior to model averaging for RRS, ranked in order of their AICc support. Models included individual ID and year as random effects.

| **a) Clutch size** | | | |
| --- | --- | --- | --- |
| Parameter | AICc | △AICc | AICc weight |
| Age | 671.22 | 0.00 | 0.38 |
| Age + F_ROH_ | 672.92 | 1.70 | 0.16 |
| Age + generation | 673.07 | 1.85 | 0.15 |
| Generation | 674.06 | 2.84 | 0.09 |
| Null | 674.17 | 2.95 | 0.09 |
| Age + F_ROH_ + generation | 674.77 | 3.55 | 0.06 |
| F_ROH_ | 676.26 | 5.04 | 0.03 |
| F_ROH_ + generation | 676.12 | 4.90 | 0.03 |
|  | | | |
| **b) Egg volume** | | | |
| Parameter | AICc | △AICc | AICc weight |
| F_ROH_ + generation | 2247.13 | 0.00 | 0.52 |
| Generation | 2247.32 | 0.19 | 0.48 |
| Null | 2259.83 | 12.70 | 0.00 |
| F_ROH_ | 2261.43 | 14.30 | 0.00 |
|  | | | |
| **c) Hatching success** | | | |
| Parameter | AICc | △AICc | AICc weight |
| F_ROH_ | 608.25 | 0.00 | 0.51 |
| F_ROH_ + generation | 610.26 | 2.01 | 0.19 |
| F_ROH_ + age | 610.30 | 2.05 | 0.18 |
| F_ROH_ + generation + age | 611.63 | 3.38 | 0.09 |
| Null | 615.30 | 7.05 | 0.01 |
| Generation | 616.97 | 8.71 | 0.01 |
| Age | 617.28 | 9.03 | 0.01 |
| Generation + age | 618.88 | 10.63 | 0.00 |

△AICc reflects the differences among selected models.

| **Trait** | Posterior Mean β (F_ROH_CHR_) | 95% Credible Interval | SD (Chromosome RE) | SD (Individual RE) |
| --- | --- | --- | --- | --- |
| **Clutch size** | -0.0007 | (-0.08, 0.07) | 0.01 | 0.23 |
| **Hatching success** | -0.0016 | (-0.13, 0.12) | 0.01 | 1.95 |
| **Egg volume** | 0.0002 | (-0.20, 0.19) | 0.02 | 3.13 |

**Supplementary Table 5.** Posterior estimates of chromosome-specific inbreeding effects (FROH_CHR) from Bayesian multi-membership models. Posterior means and 95% credible intervals reflect fixed effects of FROH_CHR. Standard deviations represent variance attributed to chromosome-level and individual-level random effects (RE). Model family for clutch size and hatching success is Poisson and Gaussian for egg volume.

**References**

Andrews, S., Krueger, F., Segonds-Pichon, A., Biggins, L., Krueger, C., & Wingett, S. (2012). *FastQC*. http://www.bioinformatics.babraham.ac.uk/projects/fastqc

Biscarini, F., Cozzi, P., Gaspa, G., & Marras, G. (2019). *detectRuns: a R Package for Runs of Homozygosity and Runs of Heterozygosity* (0.9.6). https://CRAN.R-project.org/package=detectRUNS

Broad Institute. (2019). *Picard Toolkit*. Broad Institute, GitHub Repository. https://broadinstitute.github.io/picard/

Ceballos, F. C., Joshi, P. K., Clark, D. W., Ramsay, M., & Wilson, J. F. (2018). Runs of homozygosity: Windows into population history and trait architecture. *Nature Reviews Genetics*, *19*(4), 220–234. https://doi.org/10.1038/nrg.2017.109

Danecek, P., Auton, A., Abecasis, G., Albers, C. A., Banks, E., DePristo, M. A., Handsaker, R. E., Lunter, G., Marth, G. T., Sherry, S. T., McVean, G., & Durbin, R. (2011). The variant call format and VCFtools. *Bioinformatics*, *27*(15), 2156–2158. https://doi.org/10.1093/bioinformatics/btr330

Ewels, P., Magnusson, M., Lundin, S., & Käller, M. (2016). MultiQC: summarize analysis results for multiple tools and samples in a single report. *Bioinformatics*, *32*(19), 3047–3048. https://doi.org/10.1093/bioinformatics/btw354

Foster, Y., Dutoit, L., Grosser, S., Dussex, N., Foster, B. J., Dodds, K. G., Brauning, R., Van Stijn, T., Robertson, F., McEwan, J. C., Jacobs, J. M. E., & Robertson, B. C. (2021). Genomic signatures of inbreeding in a critically endangered parrot, the kākāpō. *G3 Genes|Genomes|Genetics*, *11*(11). https://doi.org/10.1093/g3journal/jkab307

Gorssen, W., Meyermans, R., Janssens, S., & Buys, N. (2021). A publicly available repository of ROH islands reveals signatures of selection in different livestock and pet species. *Genetics Selection Evolution*, *January*. https://doi.org/10.1186/s12711-020-00599-7

Humble, E., Dasmahapatra, K. K., Martinez-Barrio, A., Gregório, I., Forcada, J., Polikeit, A. C., Goldsworthy, S. D., Goebel, M. E., Kalinowski, J., Wolf, J. B. W., & Hoffman, J. I. (2018). RAD sequencing and a hybrid antarctic fur seal genome assembly reveal rapidly decaying linkage disequilibrium, global population structure and evidence for inbreeding. *G3: Genes, Genomes, Genetics*, *8*(8), 2709–2722. https://doi.org/10.1534/g3.118.200171

Kardos, M., Taylor, H. R., Ellegren, H., Luikart, G., & Allendorf, F. W. (2016). Genomics advances the study of inbreeding depression in the wild. *Evolutionary Applications*, *9*(10), 1205–1218. https://doi.org/10.1111/eva.12414

Krueger, F. (2015). *Trim Galore: A wrapper tool around Cutadapt and FastQC to consistently apply quality and adapter trimming to FastQ files*. http://www.bioinformatics.babraham.ac.uk/projects/trim_galore/

Li, H. (2011a). A statistical framework for SNP calling, mutation discovery, association mapping and population genetical parameter estimation from sequencing data. *Bioinformatics*, *27*(21), 2987–2993. https://doi.org/10.1093/bioinformatics/btr509

Li, H. (2011b). Improving SNP discovery by base alignment quality. *Bioinformatics*, *27*(8), 1157–1158. https://doi.org/10.1093/bioinformatics/btr076

Li, H. (2011c). Tabix: Fast retrieval of sequence features from generic TAB-delimited files. *Bioinformatics*, *27*(5), 718–719. https://doi.org/10.1093/bioinformatics/btq671

Li, H. (2013). Aligning sequence reads, clone sequences and assembly contigs with BWA-MEM. *ArXiv Preprint ArXiv:1303.3997*. http://arxiv.org/abs/1303.3997

Li, H., & Durbin, R. (2009). Fast and accurate short read alignment with Burrows-Wheeler transform. *Bioinformatics*, *25*(14), 1754–1760. https://doi.org/10.1093/bioinformatics/btp324

Machado, A. P., Topaloudis, A., Cumer, T., Lavanchy, E., Bontzorlos, V., Ceccherelli, R., Charter, M., Kassinis, N., Lymberakis, P., Manzia, F., Ducrest, A. L., Dupasquier, M., Guex, N., Roulin, A., & Goudet, J. (2022). Genomic consequences of colonisation, migration and genetic drift in barn owl insular populations of the eastern Mediterranean. *Molecular Ecology*, *31*(5), 1375–1388. https://doi.org/10.1111/mec.16324

McQuillan, R., Leutenegger, A. L., Abdel-Rahman, R., Franklin, C. S., Pericic, M., Barac-Lauc, L., Smolej-Narancic, N., Janicijevic, B., Polasek, O., Tenesa, A., MacLeod, A. K., Farrington, S. M., Rudan, P., Hayward, C., Vitart, V., Rudan, I., Wild, S. H., Dunlop, M. G., Wright, A. F., … Wilson, J. F. (2008). Runs of Homozygosity in European Populations. *American Journal of Human Genetics*, *83*(3), 359–372. https://doi.org/10.1016/j.ajhg.2008.08.007

Meyermans, R., Gorssen, W., Buys, N., & Janssens, S. (2020). How to study runs of homozygosity using plink? a guide for analyzing medium density snp data in livestock and pet species. *BMC Genomics*, *21*(1), 1–14. https://doi.org/10.1186/s12864-020-6463-x

Muyas, F., Bosio, M., Puig, A., Susak, H., Domènech, L., Escaramis, G., Zapata, L., Demidov, G., Estivill, X., Rabionet, R., & Ossowski, S. (2019). Allele balance bias identifies systematic genotyping errors and false disease associations. *Human Mutation*, *40*(1), 115–126. https://doi.org/10.1002/humu.23674

Purcell, S., Neale, B., Todd-Brown, K., Thomas, L., Ferreira, M. A. R., Bender, D., Maller, J., Sklar, P., de Bakker, P. I. W., Daly, M. J., & Sham, P. C. (2007). PLINK: A tool set for whole-genome association and population-based linkage analyses. *American Journal of Human Genetics*, *81*(3), 559–575. https://doi.org/10.1086/519795

Quinlan, A. R., & Hall, I. M. (2010). BEDTools: A flexible suite of utilities for comparing genomic features. *Bioinformatics*, *26*(6), 841–842. https://doi.org/10.1093/bioinformatics/btq033

R Core Team. (2020). R: A language and environment for statistical computing. In *R Foundation for Statistical Computing*.

Wang, J., Raskin, L., Samuels, D. C., Shyr, Y., & Guo, Y. (2015). Genome measures used for quality control are dependent on gene function and ancestry. *Bioinformatics*, *31*(3), 318–323. https://doi.org/10.1093/bioinformatics/btu668
